# Risk Stratification of Acute Pulmonary Embolism and Determining the Effect on Chronic Cardiopulmonary Complications: The REACH Study

**DOI:** 10.1055/s-0040-1708558

**Published:** 2020-03-30

**Authors:** Hannah Stevens, Wendy Fang, Warren Clements, Jason Bloom, James McFadyen, Huyen Tran

**Affiliations:** 1 Department of Haematology, Alfred Hospital, Melbourne, Victoria, Australia; 2 Department of Medicine, Monash University, Melbourne, Victoria, Australia; 3 Atherothrombosis and Vascular Biology Program, Baker Heart and Diabetes Institute, Melbourne, Victoria, Australia; 4 Department of Radiology, Alfred Hospital, Melbourne, Victoria, Australia; 5 Department of Surgery, Monash University, Melbourne, Victoria, Australia; 6 Department of Cardiology, Alfred Hospital, Melbourne, Victoria, Australia

**Keywords:** pulmonary embolism, risk stratification, venous thromboembolism, pulmonary hypertension, thrombosis

## Abstract

**Introduction** Patients with acute pulmonary embolism (PE) are at risk of developing chronic complications including the post-PE syndrome with reduced cardiopulmonary function and chronic thromboembolism pulmonary hypertension (CTEPH). Risk stratification at PE diagnosis is an important tool in predicting early mortality; however, its use in predicting chronic complications has not been evaluated.

**Objective** This study investigates the effect of initial risk stratification of intermediate risk and standard risk PE on the rate of development of chronic complications including right ventricular (RV) dysfunction, residual perfusion defects, and CTEPH.

**Methods** Cases of acute PE ( *n* = 1,524) were identified using International Statistical Classification of Diseases and Related Health Problems, Tenth Revision, Australian Modification discharge diagnosis coding for PE. Evidence of RV dysfunction and systolic blood pressure < 90 mm Hg were used to risk stratify into high, intermediate and standard risk PE.

**Results** There were 508 patients included in the analysis. Intermediate risk PE was associated with higher rates of persistent RV dysfunction as well as residual perfusion defects on repeat imaging. The overall rate of CTEPH was low (0.6%) and there was no difference between the intermediate risk and standard risk PE groups.

**Conclusion** These findings demonstrate that acute intermediate risk PE is associated with higher rates of RV dysfunction on follow-up imaging than standard risk PE. However, the rate of CTEPH was similar between the two groups and overall the CTEPH rate was low among all patients with intermediate and standard risk PE.

## Introduction

Acute venous thromboembolism (VTE), including both deep vein thrombosis and pulmonary embolism (PE), is a commonly diagnosed medical condition with an incidence of approximately 1 to 1.5 per 1,000 person-years. 1 2 3 Importantly, VTE is associated with significant morbidity and mortality, and in particular acute PE is an independent predictor of reduced survival. 1 4 5 Clinical research has often focused on the evaluation of diagnosis and treatment of acute PE; however, there remains a relative lack of evidence concerning the determinants of long-term outcomes among patients with PE.

The post-PE syndrome is a recently described entity and encompasses a collection of signs and symptoms that persist following acute PE. Although no formal definition yet exists, this syndrome may include components such as suboptimal cardiac function, pulmonary artery flow dynamics or pulmonary gas exchange in combination with persistent dyspnoea, reduced exercise tolerance and diminished quality of life, without an alternative explanation. 6 Prospective research has demonstrated that 44% of patients suffering from an acute PE are described having a New York Heart Association (NYHA) heart failure score of II or higher at least 6 months after PE diagnosis. 7 Furthermore, nearly half of all patients have exercise limitation at 1-year post-PE diagnosis as measured by peak oxygen uptake on cardiopulmonary exercise testing. These functional limitations significantly impact those affected with a reduction in health-related quality of life. 8

In addition to the post-PE syndrome, the most notorious chronic complication of acute PE is chronic thromboembolic pulmonary hypertension (CTEPH). CTEPH is defined by residual thrombus on radiological imaging and evidence of raised pulmonary pressures on right heart catheterization. 9 The incidence of CTEPH is estimated to be between 0.4 and 9.1% in the 2 years following an acute PE, 10 11 12 13 14 15 16 but at present it is difficult to determine which patients will develop this debilitating disease. Medical risk factors that may predispose to the development of CTEPH include a larger initial perfusion defect, prior VTE, presence of anti-phospholipid antibodies or prior splenectomy. 10 17 18 19 The prompt recognition of CTEPH and instigating early treatment is a critical factor in its management since it remains a potentially ‘curable’ condition by way of a pulmonary endarterectomy, which can dramatically improve symptoms of dyspnoea. 20 21 This is underscored by the fact that if CTEPH remains undiagnosed or untreated, the 5-year survival is approximately 30% and is associated with a marked increase in morbidity and a detrimental effect on quality of life. 22

## Methods

### Study Design

A retrospective, observational study conducted at a tertiary referral institution. Ethics approval was obtained from the local human research ethics committee (project number 90/18).

### Population

All patients receiving inpatient management for acute symptomatic PE between January 2012 and July 2017.

### Eligibility Criteria

**Fig. 1:**
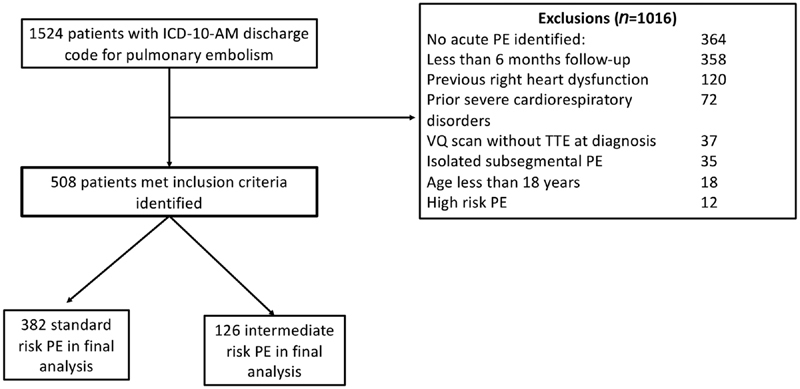
Flowchart for inclusion and exclusion criteria.

All patients with a diagnosis of acute PE treated at our centre were eligible for inclusion. Patients were excluded if: age < 18 years, known prior right ventricular (RV) dysfunction or conditions judged by the investigators to cause RV dysfunction from severe prior cardiopulmonary pathology. Additionally, we excluded isolated sub-segmental PE or high-risk PE (PE with systolic blood pressure less than 90 mm Hg for 15 minutes or more and RV dysfunction). Importantly, all patients with less than 6 months' follow-up were excluded, except those who underwent earlier radiological imaging showing no evidence of residual PE. A flowchart of the study population is shown in Fig. 1 .

### Identification of Cases

Hospital discharge records were interrogated using the International Statistical Classification of Diseases and Related Health Problems, Tenth Revision, Australian Modification (ICD-10- am ) codes corresponding to the diagnosis of acute PE.

### Risk Stratification

Following initial identification, electronic medical records, radiology reports and transthoracic echocardiogram (TTE) results were then reviewed by the study investigators to confirm the diagnosis and classify acute PE into standard, intermediate and high-risk PE according to criteria adapted from the European Society of Cardiology's *Guidelines on the diagnosis and management of acute pulmonary embolism* shown in Table 1 . 23

**Table 1:** Risk stratification of acute pulmonary embolism 23

Risk	Shock or hypotension	Right ventricular dysfunction
Standard	Absent	Absent
Intermediate	Absent	Present
High	Present	Present

### Definitions for Right Heart Dysfunction and CTEPH

RV dysfunction was defined as either:

1. TTE measurements of RV size using RV basal diameter or RV systolic dysfunction using tricuspid annular plane systolic excursion from the American Society of Echocardiography and European Society of Cardiovascular Imaging *Recommendations for cardiac chamber quantification by echocardiography in adults* . 24
2. Computed tomographic pulmonary angiography (CTPA) demonstrating a right ventricle to left ventricle ratio of greater than 0.9 on radiologist review. CT criterion was only used if TTE was not performed at diagnosis.

The diagnosis of suspected pulmonary hypertension was based on TTE using tricuspid regurgitation velocity. Confirmed CTEPH was diagnosed on right heart catheterization using the European Society of Cardiology and European Respiratory Society *Guidelines for the diagnosis and treatment of pulmonary hypertension* . 25

### Follow-up

Cases were followed for at least 6 months. Repeat radiological imaging or TTE was performed at physician discretion based on patient symptoms. Chronic complications reviewed on follow-up imaging included persistent RV dysfunction, residual thrombus and CTEPH.

### Statistical Methods

Data was collected using Excel. Statistical significance was evaluated using Fisher's exact test and unpaired *t* -test, with a *p* -value of < 0.05 regarded as statistically significant throughout.

## Results

Initial review of all discharge diagnoses of acute PE using the ICD-10- am classification identified 1,524 patients. Of these, 508 were eligible for inclusion in the final analysis ( Fig. 1 ). The general characteristics, treatment and follow-up for both groups are shown in Table 2 . The median age, sex, weight and prior history of VTE were similar between the two groups. CTPA was used to establish the diagnosis of acute PE in the majority of patients in both the intermediate and the standard risk PE groups (95% vs. 96%, respectively) and the remainder of the patients were diagnosed using ventilation/perfusion lung imaging. Intermediate risk PE was associated with an increase in proximal extent of thrombus, with 48% of patients having thrombus in the saddle or main pulmonary arteries, in contrast to 19% in the standard risk group. TTE at diagnosis was performed in 82% of the intermediate risk group in comparison to 28% of the standard risk group.

**Table 2:** Baseline characteristics of study population

	Intermediate risk PE ( *n* = 126)	Standard risk PE ( *n* = 382)	*p* -Value
Demographics			
Age, y			0.08
Median	64	60	
Interquartile range	52–74	44–71	
Female sex, (%)	56 (44)	163 (43)	0.76
Body weight, kg			0.07
Median	86	80	
Interquartile range	74–100	68–92	
Prior history of VTE (%)	25 (20)	54 (14)	0.16
VTE treatment			
Initial treatment for VTE (%)			
LMWH	109 (87)	299 (78)	0.05
UFH	13 (10)	42 (11)	1.0
Rivaroxaban	4 (3)	31 (8)	0.07
Apixaban	0	3 (< 1)	1.0
Other	0	7 (2)	0.20
Maintenance treatment (%)			
Warfarin	54 (43)	161 (42)	0.92
Rivaroxaban	48 (38)	141 (37)	0.83
Low molecular weight heparin	14 (11)	45 (12)	1.0
Apixaban	10 (8)	23 (6)	0.53
No anticoagulation	0	9 (2)	0.12
Dabigatran	0	2 (< 1)	1.0
Missing data	0	1 (< 1)	1.0
Treatment duration in months, median	5.8	5.1	0.11
Systemic thrombolysis, (%)	2 (1.5)	0	0.06
Imaging			
CTPA at diagnosis (%)	368 (96)	120 (95)	0.60
TTE at diagnosis (%)	106 (28)	103 (82)	**0.0001**
Follow-up			
Follow up in mo (median)	26.1	25.2	0.48

Abbreviations: CTPA, computed tomographic pulmonary angiography; LMWH, low molecular weight heparin; PE, pulmonary embolism; TTE, transthoracic echocardiogram; UFH, unfractionated heparin; VTE, venous thromboembolism.

Note: Bold values highlight statistically significant values.

The findings of the study are listed in Table 3 . A repeat TTE at least 3 months after acute PE diagnosis was performed in 36 (29%) patients in the intermediate risk group and 84 (22%) in the standard risk group at a median time of 10.8 months in both groups. The rate of persistent RV dysfunction identified on TTE was higher in the intermediate risk group compared with the standard risk group at 44 and 18%, respectively ( *p* = 0.003). Additionally, in the patient cohort that had repeat radiological imaging at least 3 months from diagnosis, residual perfusion defects were demonstrated in 40% of the intermediate risk group and 23% of the standard risk group ( *p* = 0.04).

**Table 3:** Study results

	Intermediate risk PE ( *n* = 126)	Standard risk PE ( *n* = 382)	*p* -Value
Suspected CTEPH (%)	2 (1.6)	1 (0.26)	0.16
95% confidence interval, %	0.08–6	0.01–1.6	
Confirmed CTEPH	0 a	0 a	–
Persistent RV dysfunction on TTE (%)	16/36 (44)	15/84 (18)	**0.003**
95% confidence interval, %	29.5–60.4	11–27.5	
Residual perfusion defects (%)	19/48 (40)	33/143 (23)	**0.04**
95% confidence interval, %	27–53.7	16.9–30.7	
Time to repeat TTE in months (median)	10.8	10.8	0.38
Time to repeat radiological imaging in months (median)	6.0	6.03	0.76

Abbreviations: CTEPH, chronic thromboembolism pulmonary hypertension; PE, pulmonary embolism; RV, right ventricular; TTE, transthoracic echocardiogram.

Note: Bold values highlight statistically significant values.

a CTEPH unable to be confirmed as patients did not undergo right heart catheterization.

Overall, the rate of suspected CTEPH in the study was 0.6%. CTEPH was suspected in 2 patients (1.6%) in the intermediate risk group and 1 patient (0.26%) in the standard risk group. However, due to medical comorbidities no patients in either group had CTEPH confirmed on right heart catheterization.

## Discussion

In this study, we have determined that following acute PE a significant proportion of patients has persistent RV dysfunction on repeat TTE, which in turn supports the notion of the post-PE syndrome. The risk of persistent RV dysfunction is increased in patients with intermediate risk PE when compared with standard risk PE (44% vs. 18%, respectively). Despite this, a diagnosis of standard risk PE is still associated with nearly one in five patients developing RV dysfunction at least 3 months after diagnosis of acute PE. These findings are supported by previous work by Stevinson et al, who demonstrated in their prospective non-interventional study that 41% of patients developed RV dysfunction or had functional impairment 6 months after a diagnosis of acute, non-massive PE. 7 This study did not, however, determine differences between standard and intermediate risk PE. Further, when evaluating the late complications from the PEITHO (Pulmonary Embolism Thrombolysis) trial, Konstantinides et al, demonstrated that 44% of patients with intermediate risk PE had persistent RV dysfunction with no difference between anticoagulation and thrombolysis. 26 Additionally, a further post hoc analysis of the PEITHO trial suggested that 13% of those with intermediate risk PE had either post-PE syndrome or CTEPH when combining both echocardiographic findings and NYHA functional classification. 27 However, direct comparison of the functional and radiological measures in these trials should be performed with caution due to differences in the definitions of outcome measures.

Our study demonstrates that initial risk stratification is useful in predicting which patients with acute PE may be at risk of developing RV dysfunction. Importantly, we have also shown that patients with standard risk PE are still at risk of developing cardiac dysfunction, thus highlighting the need for adequate long-term monitoring and follow-up in all patients with acute PE. Further prospective research will be required to understand which molecular processes lead to either the persistence of cardiac damage, or aid in initiating the development of RV dysfunction over time.

Moreover, we have demonstrated that the diagnosis of suspected CTEPH following acute PE is infrequent, with an overall rate of diagnosis of 0.6%. Previous research suggests that the size of acute thrombus impacts the development of CTEPH. 10 However, to our knowledge there is no previous data comparing the impact of initial risk stratification on chronic outcomes. We have been able to show that the rate of development of CTEPH is low in both cohorts and subsequently demonstrate that initial risk stratification does not appear to impact on the development of CTEPH.

## Limitations

This was a retrospective study and consequently there are some limitations to be addressed. First, there were no pre-specified indications for screening for CTEPH following an acute PE. As such, decisions regarding treatment and follow-up imaging or TTE was made at the discretion of the treating clinician and thus were not uniform across study participants. Moreover, as not all patients underwent subsequent lung imaging or TTE there is the potential for selection bias to occur. We acknowledge that these issues may affect the reported rates of CTEPH in the study population but importantly clinicians would perform further investigations including lung imaging and TTE if patients reported persistent dyspnoea to ensure that clinically relevant findings could be explored.

Additionally, the rate of CTEPH in both populations was lower than expected. Despite showing no significant difference in rates between the PE risk groups, it is possible that this study is underpowered given the infrequent diagnosis of CTEPH. Furthermore, as previously mentioned we excluded high-risk PE from our study due to the rarity of the diagnosis and the marked increase in mortality. As high-risk PE is often associated with larger perfusion defects in conjunction with cardiac dysfunction, it is plausible that this group of patients would have higher rates of CTEPH, but there is limited available research in this field. Given this exclusion, we cannot comment on CTEPH rates in high risk PE. However, we have been able to show that overall CTEPH rates are low in our study population and there is no role for routine screening in intermediate and standard risk PE.

Finally, the definitions for the post-PE syndrome and RV dysfunction following acute PE are continuing to develop and advance over time. Unlike CTEPH, there is no consensus on standardised diagnostic criteria which may lead to a delay in diagnosis. We believe that standardising the definitions of these conditions will aid in identifying patients with acute PE who are at higher risk of persistent RV dysfunction and will allow for close monitoring for development of CTEPH.

In summary, initial risk stratification following an acute PE can aid in predicting which patients may be at risk of persistent RV dysfunction. However, this does not appear to correlate with an increase in rate of CTEPH with overall rates remaining extremely low. These findings emphasize the importance of regular clinical review following an acute PE and highlight the need for prospective research in this area to identify high-risk patient groups and improve clinical outcomes.

## References

[1] Naess I A Christiansen S C Romundstad P Cannegieter S C Rosendaal F R Hammerstrøm J Incidence and mortality of venous thrombosis: a population-based study J Thromb Haemost 2007 5 04 692 699 17367492 10.1111/j.1538-7836.2007.02450.x

[2] Tagalakis V Patenaude V Kahn S R Suissa S Incidence of and mortality from venous thromboembolism in a real-world population: the Q-VTE Study Cohort Am J Med 2013 126 09 8.32E15 8.32E23 10.1016/j.amjmed.2013.02.024 23830539

[3] Huang W Goldberg R J Anderson F A Kiefe C I Spencer F A Secular trends in occurrence of acute venous thromboembolism: the Worcester VTE study (1985-2009) Am J Med 2014 127 09 829 3.9E6 24813864 10.1016/j.amjmed.2014.03.041 PMC4161646

[4] Heit J A Silverstein M D Mohr D N Petterson T M O'Fallon W M Melton L J III Predictors of survival after deep vein thrombosis and pulmonary embolism: a population-based, cohort study Arch Intern Med 1999 159 05 445 453 10074952 10.1001/archinte.159.5.445

[5] Søgaard K K Schmidt M Pedersen L Horváth-Puhó E Sørensen H T 30-year mortality after venous thromboembolism: a population-based cohort study Circulation 2014 130 10 829 836 24970783 10.1161/CIRCULATIONAHA.114.009107

[6] Sista A K Klok F A Late outcomes of pulmonary embolism: the post-PE syndrome Thromb Res 2018 164 157 162 28641836 10.1016/j.thromres.2017.06.017

[7] Stevinson B G Hernandez-Nino J Rose G Kline J A Echocardiographic and functional cardiopulmonary problems 6 months after first-time pulmonary embolism in previously healthy patients Eur Heart J 2007 28 20 2517 2524 17670755 10.1093/eurheartj/ehm295

[8] Kahn S R Hirsch A M Akaberi A Functional and exercise limitations after a first episode of pulmonary embolism: results of the ELOPE prospective cohort study Chest 2017 151 05 1058 1068 27932051 10.1016/j.chest.2016.11.030

[9] Simonneau G Montani D Celermajer D S Haemodynamic definitions and updated clinical classification of pulmonary hypertension Eur Respir J 2019 53 01 1.801913E6 10.1183/13993003.01913-2018 PMC6351336 30545968

[10] Pengo V Lensing A W Prins M H Incidence of chronic thromboembolic pulmonary hypertension after pulmonary embolism N Engl J Med 2004 350 22 2257 2264 15163775 10.1056/NEJMoa032274

[11] Becattini C Agnelli G Pesavento R Incidence of chronic thromboembolic pulmonary hypertension after a first episode of pulmonary embolism Chest 2006 130 01 172 175 16840398 10.1378/chest.130.1.172

[12] Klok F A van Kralingen K W van Dijk A P Heyning F H Vliegen H W Huisman M V Prospective cardiopulmonary screening program to detect chronic thromboembolic pulmonary hypertension in patients after acute pulmonary embolism Haematologica 2010 95 06 970 975 20053871 10.3324/haematol.2009.018960 PMC2878796

[13] Guérin L Couturaud F Parent F Prevalence of chronic thromboembolic pulmonary hypertension after acute pulmonary embolism. Prevalence of CTEPH after pulmonary embolism Thromb Haemost 2014 112 03 598 605 24898545 10.1160/TH13-07-0538

[14] Martí D Gómez V Escobar C Incidence of symptomatic and asymptomatic chronic thromboembolic pulmonary hypertension [in Spanish] Arch Bronconeumol 2010 46 12 628 633 20926172 10.1016/j.arbres.2010.07.012

[15] Poli D Grifoni E Antonucci E Incidence of recurrent venous thromboembolism and of chronic thromboembolic pulmonary hypertension in patients after a first episode of pulmonary embolism J Thromb Thrombolysis 2010 30 03 294 299 20157841 10.1007/s11239-010-0452-x

[16] Korkmaz A Ozlu T Ozsu S Kazaz Z Bulbul Y Long-term outcomes in acute pulmonary thromboembolism: the incidence of chronic thromboembolic pulmonary hypertension and associated risk factors Clin Appl Thromb Hemost 2012 18 03 281 288 22275389 10.1177/1076029611431956

[17] Wolf M Boyer-Neumann C Parent F Thrombotic risk factors in pulmonary hypertension Eur Respir J 2000 15 02 395 399 10706510 10.1034/j.1399-3003.2000.15b28.x

[18] Bonderman D Jakowitsch J Adlbrecht C Medical conditions increasing the risk of chronic thromboembolic pulmonary hypertension Thromb Haemost 2005 93 03 512 516 15735803 10.1160/TH04-10-0657

[19] Bonderman D Wilkens H Wakounig S Risk factors for chronic thromboembolic pulmonary hypertension Eur Respir J 2009 33 02 325 331 18799507 10.1183/09031936.00087608

[20] Taboada D Pepke-Zaba J Jenkins D P Outcome of pulmonary endarterectomy in symptomatic chronic thromboembolic disease Eur Respir J 2014 44 06 1635 1645 25234805 10.1183/09031936.00050114

[21] Olgun Yıldızeli Ş Kepez A Taş S Pulmonary endarterectomy for patients with chronic thromboembolic disease Anatol J Cardiol 2018 19 04 273 278 29615545 10.14744/AnatolJCardiol.2018.37929 PMC5998853

[22] Riedel M Stanek V Widimsky J Prerovsky I Longterm follow-up of patients with pulmonary thromboembolism. Late prognosis and evolution of hemodynamic and respiratory data Chest 1982 81 02 151 158 7056079 10.1378/chest.81.2.151

[23] Konstantinides S V Torbicki A Agnelli G 2014 ESC guidelines on the diagnosis and management of acute pulmonary embolism Eur Heart J 2014 35 43 3033 3069, 3069a–3069k 25173341 10.1093/eurheartj/ehu283

[24] Lang R M Badano L P Mor-Avi V Recommendations for cardiac chamber quantification by echocardiography in adults: an update from the American Society of Echocardiography and the European Association of Cardiovascular Imaging J Am Soc Echocardiogr 2015 28 01 1 3.9E15 25559473 10.1016/j.echo.2014.10.003

[25] Galiè N Humbert M Vachiery J L 2015 ESC/ERS Guidelines for the diagnosis and treatment of pulmonary hypertension: the Joint Task Force for the Diagnosis and Treatment of Pulmonary Hypertension of the European Society of Cardiology (ESC) and the European Respiratory Society (ERS): Endorsed by: Association for European Paediatric and Congenital Cardiology (AEPC), International Society for Heart and Lung Transplantation (ISHLT) Eur Heart J 2016 37 01 67 119 26320113 10.1093/eurheartj/ehv317

[26] Konstantinides S V Vicaut E Danays T Impact of thrombolytic therapy on the long-term outcome of intermediate-risk pulmonary embolism J Am Coll Cardiol 2017 69 12 1536 1544 28335835 10.1016/j.jacc.2016.12.039

[27] Barco S Russo M Vicaut E Incomplete echocardiographic recovery at 6 months predicts long-term sequelae after intermediate-risk pulmonary embolism. A post-hoc analysis of the Pulmonary Embolism Thrombolysis (PEITHO) trial Clin Res Cardiol 2019 108 07 772 778 30564950 10.1007/s00392-018-1405-1 PMC6584226

